# Is Eating Less Meat Possible? Exploring the Willingness to Reduce Meat Consumption among Millennials Working in Polish Cities

**DOI:** 10.3390/foods11030358

**Published:** 2022-01-26

**Authors:** Agata Szczebyło, Ewa Halicka, Krystyna Rejman, Joanna Kaczorowska

**Affiliations:** Institute of Human Nutrition Sciences, WULS-SGGW, 02-787 Warsaw, Poland; ewa_halicka@sggw.edu.pl (E.H.); krystyna_rejman@sggw.edu.pl (K.R.); joanna_kaczorowska@sggw.edu.pl (J.K.)

**Keywords:** meat consumption, meat attachment, behaviour change phases, sustainable diet

## Abstract

Reducing the consumption of meat constitutes an important part of the global shift towards more sustainable food systems. At the same time, meat is firmly established in the food culture of most human beings, and better understanding of individual behaviors is essential to facilitate a durable change in contemporary eating patterns. To determine the level and nature of attachment to meat among consumers, the Meat Attachment Questionnaire (MAQ) in relation to the phases of behaviour change in the meat consumption reduction process was utilised. Data collected through a survey carried out among Poles aged 25–40 years living in cities were analysed with the use of Spearman’s correlations and one-way ANOVA with Tukey’s post-hoc tests. The biggest share of the studied group of millennials (N = 317) never considered reducing their meat consumption (Phase 1–41%) and was described by the highest level of MAQ score in all its categories: hedonism, affinity, dependence, and entitlement. More than half of the respondents in Phase 2 participants (“planners”) declared a willingness to cut down meat consumption but had not yet put their intentions into practice. Respondents qualified in Phase 3 declared the highest willingness to reduce meat consumption and were significantly less attached to meat regarding all MAQ categories than respondents in Phase 1. The 9% of the study participants (Phase 4) had already limited the frequency of their meat consumption to “several times a week”, this however still remains insufficient compared to the ambitious goals of sustainable healthy diets. Results indicated that meat attachment categories, especially hedonism and dependence, were identified as predictors of willingness to reduce meat consumption. Research exploring the determinants of change and possibilities of effective communication about meat reduction on an individual level in different cultural settings are needed.

## 1. Introduction

The urgent need to change current dietary patterns has achieved a consensus around the world in the light of the global syndemic of obesity, undernutrition and climate change [1]. Shifting towards high consumption of plant-based foods and substantially limiting animal source foods remain a key priority in this process [2]. One of the recommendations of healthy and sustainable diets is a significant reduction of meat consumption, especially in high-income countries [3,4]. Decreased meat consumption may beneficially impact all domains of sustainability i.e., health, environment and biodiversity protection, society, economy and culture [5,6,7,8,9].

Many studies set meat in a central role in the development of humans, as a carnivorous species [10]. Initially, meat came only from hunting wild animals and could be named as an element of evolutionary heritage [11]. In this perspective livestock production is key to global food security. In fact, particularly vulnerable population groups rely on livestock in changing climates because animals have the ability to adapt to marginal climatic conditions [12]. However, globally economic and social development has led to a dynamic increase in the demand for meat, its production and the global meat trade. Today, therefore, the main source of meat is intensive and industrial livestock farming, where special farming conditions and feeding systems are used. This leads to environmental and ethical concerns. Still, as a food product, meat is a condensed source of high biological value proteins and other nutrients, among them easily absorbed heme iron, zinc, vitamin B1, B12, niacin [13,14,15]. However, it also naturally contains saturated fatty acids and cholesterol, the consumption of which should be limited due to the fact that many studies indicate them as risk factors for heart diseases [16,17,18]. High or excessive meat consumption is observed in high-income countries and it was found to be linked to a higher rate of total mortality, cardiovascular diseases, type 2 diabetes and colorectal cancer [19,20,21]. The International Agency for Research on Cancer (IARC) reported that each 50 g portion of processed meat eaten daily increases the risk of colorectal cancer by 18% [22]. One of the main recommendations for preventing cancer is to limit red and processed meat consumption [22,23]. Also, poultry meat was found to be associated with several digestive diseases [24]. Additionally, meat and meat products intake have been linked to adverse dietary patterns with insufficient amounts of vegetables and fiber, and behaviors, such as smoking and too high alcohol consumption [18,25]. 

The production of food of animal origin leads to significantly higher greenhouse gas (GHG) emissions per unit mass compared to the production of plant-based food raw materials. The results of the Global Livestock Environmental Assessment Model (GLEAM) [12] indicate that emissions from livestock supply chains represents 14.5% of global anthropogenic GHG emissions. A significant part of monocultural crops worldwide, including coarse grains, responsible (among other) for biodiversity loss and deforestation, are dedicated to feed [26,27,28]. From an environmental and social perspective, growing cereals crops for livestock feed is inefficient [29]. Domesticated animals raised in an agricultural setting consume one third of global cereal production and use about 40% of global arable land [30]. 

Growing concerns about unhealthy dietary patterns and food system sustainability are being mirrored in recommendations to reduce meat intake in developed countries. For example, the Danish dietary guidelines “good for health and climate” indicate that 350 g of meat per week is adequate in a plant-rich and varied diet and suggest especially limiting the consumption of beef and lamb [31]. In Poland, the 2020 revised dietary guidelines specify a maximum meat consumption of 500 g per week [32]. The UK official recommendations suggest that high meat consumers should reduce their intake of red and processed meat to the population’s average 70 g/day (490 g/week) [33]. The model of planetary healthy diet, developed by the EAT-Lancet Commission in 2019 set the maximum level of meat intake at 300 g per week [2]. Meat consumption in Poland significantly exceeds these recommendations. Although household budget surveys show that the consumption of meat and processed meat has decreased slightly (by 8.8%) in the second decade of the 2000s, it amounted to 1169 g/person per week in 2020. It should be noted that this figure does not include food eaten out-of-home, while expenditure on catering services almost doubled over the same period [34]. Meat consumption at the level of national food balances is 77.6 kg/person in 2020 [35], equivalent to almost 1500 g/week. 

Dietary guidelines in many countries (including Poland) advise eating more pulses and other foods that are sources of plant protein to achieve a food consumption levels that reflect a healthy sustainable diet [36,37,38]. These foods provide a sufficient set of amino acids, and likewise are a significant source of fiber, unsaturated fatty acids and valuable micronutrients [39]. Plant-based protein production utilizes far fewer natural resources and is more efficient in population sustenance [40,41]. 

Limiting meat consumption is urgent and requires changes from all the stakeholders of the food system [42]. The consumers’ responsibility cannot be underestimated especially in high-income countries, as individual decisions determine the health status of a person but also influence the market [43,44] and environment. Nutrition scientists and consumer behaviour experts strive to understand whether consumers want to eat less meat and what determines their willingness to reduce the demand for meat. In studies on the determinants of changes in individual consumer choices, much attention has been focused on personal motivations and attitudes towards eating meat. In recent years, measures have been developed and recommended for use in research on how consumers are attached to eating meat and on the opportunities in reducing meat intake. The aim of our study is to explore the attachment to meat consumption in different phases of its reducing.

## 2. Methods

### 2.1. Study Design

In our study we adapted two scales introduced in previous publications: phases of behavioral change based on Weibel et al. [45] and the Meat Attachment Questionnaire (MAQ) developed by Graça et al. [46]. Psychological phase models describe the process of behavioral change as a linear process comprising different phases and integrating the dynamic nature of human behavior. Weibel et al. proposed a self-regulation model that includes four phases that people go through when they change their behaviour: the pre-decisional (1), the pre-actional (2), the actional (3), and post-actional (4), which was adapted to study the reduction of meat consumption. The MAQ tool measures the positive bond towards meat and was developed and validated through several studies in Portugal aimed at deepening knowledge on consumer willingness to reduce meat consumption [46]. Across samples, a four-factor solution (i.e., hedonism, affinity, entitlement, and dependence) with 16 items and a second-order global dimension of meat attachment fully met criteria for good model fit. MAQ has been used in different studies, e.g., exploring New Zealand consumers’ motivations and attitudes to meat consumption [47], measuring the meat paradox among Australians [48], investigating German consumers’ preferences for meat and plant-protein blends products [49] and parental meat attachment and meat reduction in children’s diets [50]. 

The attitudes of Polish 25–40 year-olds towards meat consumption was explored from the perspective of self-assessment variables and multidimensional background of attachment to meat. The study group was chosen due to the long-term nature of the effects of behaviour and dietary changes in younger adults. In a broader perspective, the choices of employees living in city influence the market and shape the food system as a whole, and at family level, as parents of small children, millennials, influence the formation of tastes and eating patterns of the next generation. Moreover, new consumer trends, including food and nutrition, spread from the inhabitants of large cities to the rural population, for which they constitute a certain model of future food consumption and behaviour [51]. The following four research questions were investigated in the study and elaborated on in this paper:

A. What phase of change in terms of reducing meat consumption are the respondents in?
B. Does willingness to reduce meat intake depend on socio-demographic variables?
C. Is meat consumption frequency linked to the respondents’ self-assessment phase of change?
D. Are behavioral phases linked to total MAQ scores and scores in each of the four categories of attachment?

### 2.2. Participants

The survey data were collected using the computer-assisted web interview (CAWI) method in March 2019. The questionnaire was distributed online among members of a consumer panel by a commercial market research company. Participants for the current study was recruited among individuals from another study published by our research team [52]. Two criteria for participation in the survey were established: age between 25–40 years and working in the city. The definition of “work” was adopted as the provision of continuous work based on an employment contract, or in the form of a civil law contract, or a self-employed business activity. People following a meatless diet were excluded from the study. The research agency directed renumeration to the respondent’s panel account only after the questionnaire was properly completed. The survey was approved by the WULS-SGGW Ethics Committee of Scientific Research with the Participation of People (approval code 13p/2018).

Ultimately, 317 people took part in the study, including 161 women and 156 men, aged 25–40, working in cities in all 16 voivodships of Poland. The sample was of controlled quota type and was representative in terms of age and gender, based on demographic data from the national statistical office Statistics Poland (GUS). More than half (52%) of participants lived in towns and cities up to 100,000 residents, 27% in cities of 100–500,000 residents and 21% in the biggest Polish cities with more than 500,000 residents. White-collar workers consisted 70% of a sample, and blue-collar were 21%, 9% did not answer the question. The vast majority (82%) were employed under an employment contract, while 18% were employed on different kinds of contracts or self-employed. It was determined that 32% of the sample lived in three-person households, 24% in two-person households, 24% in four-person households, 12% lived alone, and 8% lived in households of five persons or more.

### 2.3. Questionnaire

The questionnaire covered many topics, not all of which were directly relevant to this paper. The survey’s parts used in this paper focused on meat-eating habits. The Meat Attachment Questionnaire was translated into Polish and verified by a bilingual speaker.

### 2.4. Measures

#### 2.4.1. Frequency of Meat Consumption 

To measure the frequency of meat consumption, a meat products index (MPI) was calculated as the arithmetic mean of the responses to two questions: the frequency of meat consumption and the frequency of meat product consumption (i.e., cold cuts, sausages, frankfurters, pates) (see Table 1, Research Question B).

#### 2.4.2. Phases of Change of Meat Consumption

All participants, when indicating the statement relevant to them regarding the degree of change in their behaviour towards meat, fell into one of four phases of change. Phase 1 grouped typical meat consumers who agreed that they never considered reducing their meat consumption (so-called “regulars”). Phase 2 participants (“planners”) declared that they had considered reducing their meat consumption but had not yet put their intentions into practice. Phase 3 respondents (“testers”) stated that “I make sure I consume less meat occasionally. In the future it is my firm intention to do this on a regular basis”. Those who indicated that they “take consuming little or no meat for granted” were grouped in Phase 4 (and labelled “reducers”). 

#### 2.4.3. Willingness to Limit Meat Consumption

To verify the self-reported assessment of change, we asked whether respondents wanted to limit their meat consumption using a 5-point Likert scale from “1—definitely do not want”, to “5—definitely want”. Then, we correlated the results with the phases of change of meat consumption. 

#### 2.4.4. Meat Attachment Questionnaire (MAQ) 

In this part of the questionnaire, respondents indicated their level of agreement with 16 statements representing four categories of reasons for attachment to eating meat. Scores of each group of statements were averaged to create a category score, while all statements were averaged to create a total scale score. 

The hedonism category included four statements, where higher scores (following the Likert scale, see Table 1) indicated pleasure in eating meat: H1: To eat meat is one of the good pleasures in life, H2: I love meals with meat, H3: I’m a big fan of meat, H4: A good steak is without comparison. 

The affinity category included four statements with reversed scores, as the category was measured as opposed to feeling of repulsion: A1: I feel bad when I think of eating meat, A2: To eat meat is disrespectful towards life and the environment, A3: Meat reminds me of diseases, A4: By eating meat I’m reminded of death and suffering of animals. In result, higher scores indicated affinity towards meat consumption. 

The entitlement category included three statements describing human privilege to eat meat: E1: According to our position in the food chain, we have a right to eat meat, E2: To eat meat is an unquestionable right of every person, E3: Eating meat is a natural and indisputable practice. 

The dependence category included five statements, which indicated feelings of dependence on meat: D1: Meat is irreplaceable in my diet, D2: I would feel fine with a meatless diet (reversed score), D3: If I couldn’t eat meat, I would feel weak, D4: If I was forced to stop eating meat, I would feel sad and D5: I can’t picture myself not eating meat regularly. 

### 2.5. Statistical Analysis

All statistical analyses were performed using the SPSS Statistics software package version 26 (SPSS Inc., Chicago, IL, USA). The Spearman’s correlation was chosen to estimate the association between the created MPI and the frequency of meat consumption (rho = 0.89; *p* < 0.001), and respectively between MPI and frequency of meat products consumption (rho = 0.92; *p* < 0.001). Differences between respondents classified in the four phases and other variables were analysed using one-way ANOVA, with Tukey’s post-hoc tests. The internal reliability for the four MAQ categories was checked by the Cronbach’s alpha coefficient and was amounted to 0.882 (hedonism), 0.891 (affinity), 0.836 (entitlement), 0.851 (dependence).

## 3. Results

### 3.1. Frequency of Meat Consumption

#### Phases of Change

The collected data showed that all four phases of behaviour change related to eating meat were represented in the study group. The biggest share, 41% (*n* = 129) of the sample was in Phase 1 (Ph1). Respondents in Phase 2 (Ph2) constituted 27% (*n* = 87) of the sample, 23% (*n* = 73) of individuals were in Phase 3 (Ph3) and 9% (*n* = 28) in Phase 4 (Ph4).

**Figure 1:**
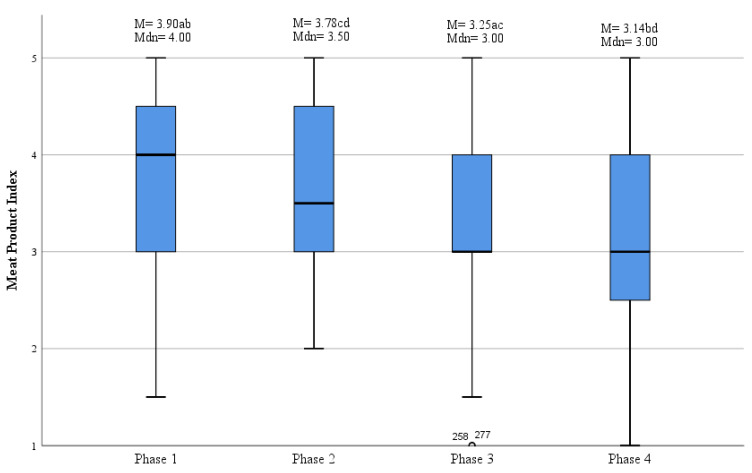
Meat Products Index distribution among respondents in phases of change in meat consumption. M—mean, Mdn—median. The values with different superscripts indicate significant differences in Tukey’s post-hoc test results (*p* < 0.001). Designation of frequency scale: 5—several times a day, 4—once a day, 3—several times a week, 2—once a week, 1—1 to 3 times a month.

The meat products index and the declared phase of change in meat consumption were significantly correlated (Figure 1). Respondents in Ph1 and Ph2 (i.e., those who did not limit the amount of meat they eat) consumed meat more often compared to those in Ph3 and Ph4. Regulars (Ph1) ate meat most often, while testers (Ph4) least frequently. Still, the average frequency of eating meat in the studied group was 3.65, which indicates almost “once a day” and even among reducers (Ph4) the MPI was 3.14, which is “several times a week”.

### 3.2. Willingness to Limit Meat Consumption

**Figure 2:**
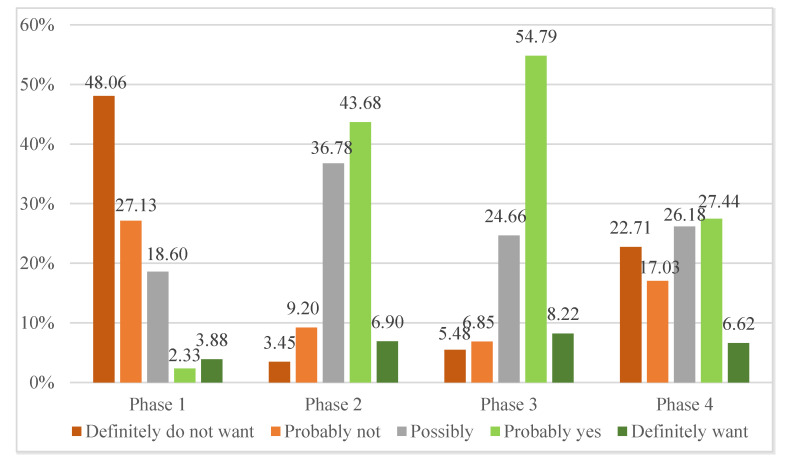
Willingness to limit meat in the diet depending on phase of change in meat consumption.

More than 75% of regulars (Ph1) did not want to reduce the amount of meat they eat (Figure 2). On the other hand, more than half of the planners (Ph2) and 63% of testers (Ph3) had the opposite intention. Opinions among reducers (Ph4) turned out to be most evenly distributed. Almost 40% of them did not want to limit their average meat consumption, while 34% did. The average values of the willingness to limit meat consumption were: Ph1 = 1.81; Ph2 = 3.14; Ph3 = 3.53; Ph4 = 3.03. The analysis confirmed significant differences in the willingness to decrease meat consumption between respondents in Ph1 and other phases (*p* < 0.001).

### 3.3. Socio-Demographic Variables

No relationship was found between phases of meat consumption reduction and the gender of respondents (*p* = 0.234) and there was also no relationship between phases and household size (*p* = 0.324). However, both characteristics determined the frequency of meat consumption (MPI). Males consumed meat more frequently than females (*p* = 0.046). The MPI for men was 3.77 and for women 3.54. The frequency of meat consumption increased with household size (*p* = 0.009). In one-person households, the MPI was 3.35, while in households of 4 or more people it was 3.89.

### 3.4. MAQ Factors

**Figure 3:**
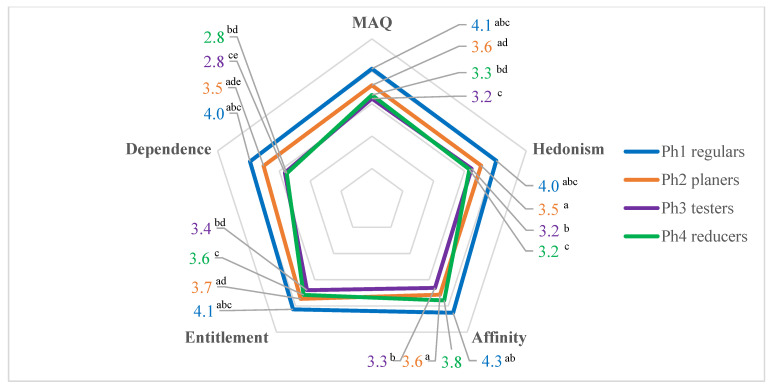
Phases and Meat Attachment Questionnaire (MAQ) results (5-point Likert scale: definitely not (1)—definitely yes (5) for total MAQ and individual categories; The values with the same superscript letters in a total and individual MAQ categories are significantly different in Tukey’s post-hoc test results (*p* < 0.001).

Significant differences between phases of change in meat consumption and MAQ categories were observed. As expected, the highest MAQ scores overall and in each category, i.e., hedonism, affinity, entitlement, dependence, were noted among consumers who declared regular meat consumption (Ph1) (Figure 3). Each statement in the hedonism-linked category was rated significantly higher by respondents in Ph1 compared to those in the other three phases. Also in Ph1, the entitlement category statement (“humans have the right to eat meat, according to the position in a food chain”) scored the highest mean (4.4) and was significantly higher than in the other phases. 

Regulars (Ph1) strongly agreed that meat is irreplaceable in their diet (mean 4.3) as well as that they cannot picture themselves not eating meat regularly (mean 4.1), which influenced the highest level of dependence category score compared to other phases. However, the reducers (Ph4) indicated the dependence opinions at the lowest level (mean 2.8) among all statements of the questionnaire. Consequently, testers and reducers (Ph3 and Ph4) were described by the lowest scores in the dependence category (2.8 equally). The highest scores (mean 4.3) in all statements in the affinity category (with reversed scores) were obtained by regulars (Ph1). Surprisingly, reducers (Ph4) had the second highest mean score (3.8) in the affinity category. 

## 4. Discussion

This paper points to a strong attachment to meat among people aged 25–40 years working and living in Polish cities, which is a predictor of resistance (low level of willingness) to cut back meat consumption. Respondents who intend to or have already reduced their meat consumption were driven by different levels of meat attachment factors compared to those with no willingness to change (and the differences were significant).

In recent years excessive meat intake has become a more controversial issue due to social, environmental and ethical reasons. Despite extensive discussion and scientific evidence on the adverse consequences of eating meat, it is still widely consumed. People choosing to eat meat are often conflicted by the “meat paradox” [53,54,55]. They confront two opposite emotions—compassion towards animals suffering from farm and industry practices and the pleasure and habit of eating meat as an essential and even staple food in the everyday diet. Therefore, they commonly develop rationalisation as a mechanism that morally justifies their decisions. Rationalisation can be described as a belief that eating meat is natural, normal, necessary and nice; the so-called 4Ns [56]. This approach can be compared with categories in MAQ. “Niceness” relates to the taste of meat which in common opinion is satisfying and brings pleasure, so it links to the hedonism category. Describing eating meat as “natural” refers to the biological hierarchy and the human position in evolution, which is the entitlement category in MAQ. “Necessary” appeals to the requirement of eating meat for health, so it compares to dependence category. Finally, affinity category in MAQ can be described as normative behaviour constructed by societal norms. Most people do not feel repulsion towards meat and do not link diseases with this food because they have been brought up to think that meat is a “normal”, culturally accepted food. In the cited study all 4Ns were endorsed at the strongest level by omnivores and those individuals, who do not tend to decrease their intake of food of animal origin. Likewise, in our study, regular meat eaters in Ph1 had the highest MAQ scores. The entitlement category, together with affinity was the most accepted set of beliefs across participants in different phases, although there were still significant differences mainly between regulars and participants in other phases. This is in line with Piazza et al. [56], that the naturalness of eating meat by humans is the most consistent factor across omnivores and restricted omnivores, which means that it is the widely accepted belief. Moreover, in our study the high score in the affinity category for regulars as well as for reducers can be linked with the central position of meat in Polish culinary culture [13,57] and in agricultural production (Poland is the largest poultry meat producer in the EU, supplying 20% of total production, it also supplies 9% (fourth place) of pork production [58]). As a result, this can suggest that restrictions in meat consumption can be driven by factors other than repulsion.

It was also shown in our study that the dependence category divided consumers who declared regular meat consumption (Ph1) and those who evolved towards limiting meat intake (Ph3 and Ph4). The dependence category was related to the perception of meat being good for health. This link was shown also in other studies, where meat eaters were less likely to believe that meatless food choices are nutritionally adequate [59]. Health concerns regarding eating meat can be seen twofold—as a potential risk factor for diseases (as cardiovascular disease or cancer) when consumed excessively, or as a fundamental source of protein needed for proper body functioning. The first aspect itself can be insufficient when it comes to behavioral changes, as among vegetarians ethical and environmental concerns additionally play a role, and consistent meat reducers take the cost of meat and weight control more often into account [47,60]. The second aspect of health concerns seems to be more present in our Ph1 group, which correlates more with a meat-eating justification attitude. Testers and reducers however, were significantly less dependent on meat consumption. This change of attitude can be linked to knowledge about non-meaty sources of nutrition compounds, especially protein, but also to increased acceptance of taste of plant-based dishes [61], which was related with cooking skills and knowledge about dishes prepared from non-animal products [52,62]. Also other researchers stated [63] that the key to communicating the needed switch towards meat reduction lies in recipe knowledge and culinary habits. Consumers need to recognise and expand their repertoire of dishes to include new full meals in which meat is replaced or partially substituted by alternative proteins, e.g., pulses, soy products or nuts. As was shown in the experiment in canteens, the partial replacement of meat in a dish can be introduced without a drop in taste acceptance [64]. Various interventions, including nudges techniques, can be implemented in food service establishments, which can be helpful in implementing new meals in consumers’ perception [65], as an established architecture of choice where cultural habits are maintained can be seen as one of the main barriers in the process of decreasing animal-based protein consumption [66]. 

One of the most relevant findings of our study is that there is a very different approach towards decreasing meat consumption among participants in each phase of behavioral change. Those individuals who declared eating little or no meat on daily basis, admitted eating meat at least several times a week. On the other hand, another study showed that consumers who considered themselves meat-eaters could restrict their meat consumption even to 1–2 times a week [67], which could easily classify them into a meat-reducer group or even semi-vegetarian or flexitarian [68]. Comparably, Neff et al. [69] defined meat reducers as those who declared eating less meat than three years earlier. This relates to much lower willingness to further meat reduction among reducers (Ph4) than testers (Ph3) who are in the actional phase of transition. Those who perceive their meat consumption as already reduced are less likely to make more effort towards further change. In Poland there is a group of consumers who traditionally refrain from eating meat products at least once a week due to religious customs, but they may be rather unaware of other motives for meat intake reduction, which are better recognized by the younger generation [67,70]. It seems relevant in further research to describe more accurately the magnitude of meat consumption, both in terms of frequency and quantity. Low meat eaters or flexitarians could be defined as those who eat up to 50 g of meat per day [2,29]. 

The respondents in our study were young people in their 20s and 30s. Making life-changing decisions that impact their lives for many years ahead is very common in this age group and may result in change of a lifecycle stage, which influences the attitudes towards food [71]. With the arrival of a child, the household acquires eating habits that solidify and shape the child’s habits and parents’ attachment to meat was shown to be crucial in a meal choice for a child [50]. In our study, meat consumption grew as the household expanded. In Poland, pork and poultry meat are cheap and preparing meat meals is perceived in terms of convenience [72,73]. At the same time, studies showed, that parents do not engage their children in discussions about the need to limit the consumption of meat and other animal products for environmental reasons [74]. In our study the MPI in one-person households was 3.35, which implies that singles may be more aware of the sustainability challenges of today’s food consumption pattern. Regarding other demographic characteristics, meat consumption in men was more common than in women participating in our study, which is in line with many other studies reviewed in 2019 by Graca et al. [70]. Research also showed that mammalian meat is positively associated with maleness [75] and that women have significantly more positive attitudes towards vegetarian and vegan diets [76]. In Poland, meat was rationed in the 1980s due to commodity shortages. However, many people refer with nostalgia to the times when food was perceived as being of better quality. It is a widely accepted fact that people in Poland once consumed meat occasionally, and products such as beans and peas were present in meals far more often than today. Based on FAO Food Balance Sheets the availability of meat for consumption in Poland surpassed 1.7 kg/week in 2018 (the EU average was 1.5 kg per week) (FAO, 2021). At the same time, in 2019 49% of Poles were concerned by antibiotic, hormone or steroid residues in meat [77]. Distrust of modern methods of animal husbandry and food production among people interested in healthy food was also described [78]. Those concerns, if properly shifted into more fact-based knowledge about food systems and nutrition could result in a combination of traditional and modern attitudes to Polish diets with lower amounts of higher-quality meat. 

Transitions in dietary behaviors requires behavioral facilitation and new value creation [79]. Changing dietary behavior at such a deep level of belief is very difficult. Messages focused on health or environmental issues had limited impact on behavior change, especially for those who do not declare similar concerns about eating meat [80,81]. Environmental concerns in particular were found to have a limited contribution in the behaviour change process [82]. However, they have the potential to influence consumers that are in transition, as the planners and testers in our study. Based on the collected data, we could conclude that messages focused on the pleasure of plant-based meals could result in their higher acceptance level. Despite the limited evidence on the effectiveness of such a framing shown by Vaillancourt C., et al. [83], this strategy still seems promising. The question who should ultimately be responsible for conducting meat reduction interventions remains relevant. Until a few years ago, due to the difficulty of changing individual behaviors and lifestyles, even environment-focused NGOs did not address the issue of limiting meat consumption in their main activities. [84]. Currently, this topic is being approached by global organisations including the UN [85] or WWF [86]. The European Union’s “Farm to Fork Strategy” highlights the need to implement different strategies, which will result in improved animal husbandry conditions and the supply of better quality and more expensive meat to the market. The economic barrier can be expected to force consumers to make more sustainable food choices [87]. Consumer market pull has a huge potential impact on production and other elements of the food system. 

### Limitations

The present study has several limitations. Meat consumption was based on self-reported frequency of consumption so it might differ from an objective measure of actual meat intake due to individual concerns regarding social acceptance of this food group. Indicating the precise amount of meat consumed daily or weekly is crucial to define what meat reduction really means to consumers. The study sample was limited due to funding constraints to the respondents of a specific age and living in cities. We recommend further research in broader population groups, including inhabitants of rural areas in Poland and other countries. 

## 5. Conclusions

In order to explore the process of change and willingness to limit meat consumption it is important to understand the socio-cultural background of consumer behaviour. 

This study indicates that strong attachment to meat and especially its two categories—hedonism and dependence—can be predictors for a low level of willingness to decrease meat consumption among Polish millennials. However, the amount of meat consumed (even in the case of reduced amounts) is still high compared to the goals of a sustainable diet. The environmental and health domains of the concept share common aims, but social and cultural domains seem to remain in opposition to the necessary changes. There is a growing discussion in Europe about climate change, including the harmful effects of agriculture and, in particular, livestock production and consumption on planetary health. Knowledge about this topic and its impact on dietary behaviour is limited and requires further research in other population groups and countries where meat consumption is very high. The MAQ was found to be a useful tool for exploring meat attachment and when compiled with other measures, can provide profound research results that can further be used to develop sustainable food policy activities.

Further research should include a more accurate description of the level and structure of meat consumption, both in terms of frequency and quantity, bridging together the MAQ tool with other measures in other population groups, including youth and rural inhabitants.

## Figures and Tables

**Table 1:** Operationalization of research questions: survey questions and measurement.

Research Question	Variables	Measurement Level and Type
A. In what phase of reducing meat consumption are the respondents in (self-assessment)?	*Dependent variable * Phase Model (PM) *Descriptive factor* Willingness to limit meat consumption	Phase 1: *I have never considered reducing my meat consumption.* Phase 2: *I’ve considered reducing my meat consumption, but I haven’t yet put this plan into practice.* Phase 3: *I make sure I consume less meat occasionally. In the future it is my firm intention to do this on a regular basis. * Phase 4: *I take consuming little or no meat for granted.* *Q: Indicate your willingness to limit your consumption of meat on a scale from 1—definitely do not want, to 5—definitely want.*
B. What is the frequency of eating meat and meat products in each phase?	Meat Products Index (MPI)	The mean of the answers to two questions: *Q1: How often do you usually eat meat?* *Q2: How often do you usually eat meat products, i.e., cold cuts, sausages, frankfurters, pates?* *Nominal scale: several times a day (5), once a day (4), several times a week (3), once a week (2), 1–3 times a month (1)*
C. Does willingness to reduce meat intake depend on socio-demographic variables?	*Socio-demographic variable* Gender Size of household	Nominal: male, female Nominal: 1, 2, 3, 4, ≥5 people
D. Are phases linked to the respondents’ total MAQ scores and each category score?	16 statements from the Meat Attachment Questionnaire grouped into four categories (hedonism, affinity, entitlement, dependence)	*Q: Please indicate to what extent you agree with the following statements-five-point Likert scale: definitely not (1)–definitely yes (5)*

## Data Availability

The data presented in this study are available on request from the corresponding author. The data are not publicly available due to privacy restrictions.
